# Improving access for community health and sub-acute outpatient services: protocol for a stepped wedge cluster randomised controlled trial

**DOI:** 10.1186/s12913-016-1611-3

**Published:** 2016-08-09

**Authors:** Katherine E. Harding, Jennifer J. Watts, Leila Karimi, Mary O’Reilly, Bridie Kent, Michelle Kotis, Sandra G. Leggat, Jackie Kearney, Nicholas F. Taylor

**Affiliations:** 1 La Trobe University, Kingsbury Drive, Bundoora, VIC 3086 Australia; 2 Eastern Health, 5 Arnold Street, Box Hill, VIC 3128 Australia; 3 Deakin University, 221 Burwood Highway, Burwood, VIC 3125 Australia; 4 Victorian Department of Health and Community Services, 50 Lonsdale Street, Melbourne, VIC 3000 Australia; 5 Plymouth University, Drake Circus, Plymouth, Devon PL4 8AA UK

## Abstract

**Background:**

Waiting lists for treatment are common in outpatient and community services, Existing methods for managing access and triage to these services can lead to inequities in service delivery, inefficiencies and divert resources from frontline care. Evidence from two controlled studies indicates that an alternative to the traditional “waitlist and triage” model known as STAT (Specific Timely Appointments for Triage) may be successful in reducing waiting times without adversely affecting other aspects of patient care. This trial aims to test whether the model is cost effective in reducing waiting time across multiple services, and to measure the impact on service provision, health-related quality of life and patient satisfaction.

**Methods/design:**

A stepped wedge cluster randomised controlled trial has been designed to evaluate the impact of the STAT model in 8 community health and outpatient services. The primary outcome will be waiting time from referral to first appointment. Secondary outcomes will be nature and quantity of service received (collected from all patients attending the service during the study period and health-related quality of life (AQOL-8D), patient satisfaction, health care utilisation and cost data (collected from a subgroup of patients at initial assessment and after 12 weeks). Data will be analysed with a multiple multi-level random-effects regression model that allows for cluster effects. An economic evaluation will be undertaken alongside the clinical trial.

**Discussion:**

This paper outlines the study protocol for a fully powered prospective stepped wedge cluster randomised controlled trial (SWCRCT) to establish whether the STAT model of access and triage can reduce waiting times applied across multiple settings, without increasing health service costs or adversely impacting on other aspects of patient care. If successful, it will provide evidence for the effectiveness of a practical model of access that can substantially reduce waiting time for outpatient and community services with subsequent benefits for both efficiency of health systems and patient care.

**Trial registration:**

Australian and New Zealand Clinical Trials Registry ACTRN12615001016527. Approved 15/9/2015.

## Background

Many patients face long waiting times for outpatient and community health services, potentially leading to physical deterioration and reduced engagement in services [1–4]. Such waits have also been associated with increased anxiety and decreased levels of community participation [5, 6]. Strategies most commonly used for managing waitlists and reducing waiting time in health services are often ineffective, or are successful only under specific circumstances. Injections of resources to temporarily boost supply of health services without a subsequent change in service delivery often have only short-term effects, with waiting lists tending to recur over time. The Netherlands provides a case in point, where $3 billion was made available in addition to normal health funding for waiting list reductions in 1999–2001, and had no impact on waiting list reduction. The same number of people were still waiting for care five years later [7]. Increasing resources in isolation are not sufficient to affect waiting list reductions; substantive changes to the delivery of services are required.

Triage systems that place new patients onto a waitlist and then use protocols to guide decisions about who should be seen next are a common strategy across a wide range of outpatient services [8] but are not always effective in improving patient flow. While triage systems may have an important role in discriminating between patients who require health care services and those who do not [9] and prioritising those who need urgent care [10], they also have drawbacks. The assignment of triage categories is often unreliable [11, 12], and triage systems can lead to inefficiencies in service delivery by diverting resources from frontline care inadvertently contributing to waiting time [13]. Furthermore, a study of the triage system in community rehabilitation in a large metropolitan health service showed that the system made little difference to waiting time for the vast majority of patients [14].

There are several specific elements in access and triage processes that have been shown to reduce waiting time and improve patient flow. For example, a recent review of the literature found that the ability to manage less resource intensive cases and/or commence initial management at triage was effective by providing the opportunity to quickly address simple needs on identification rather than placing a patient on a waiting list to be reassessed in the future [8]. Commencing initial management at triage requires triage to be conducted by somebody close to the face of service provision, and has been used successfully in emergency departments [15, 16], outpatient clinics [17] and mental health services [3].

Another important consideration in maximising patient flow is to identify whether there is a true imbalance between supply and demand, and to target interventions accordingly. Some waitlists are stable over time, indicating that the number of referrals being received must roughly equal the number of patients being discharged, but a constant backlog leads to a constant delay. Triage systems are then used to sort the patients who are waiting according to urgency. The Advance Access system designed for primary care used an initial injection of resources to manage the backlog followed by a system level change to maintain patient flow [18], and has been shown to reduce the time to see a general practitioner from several weeks to 1 or 2 days in clinics with previously long waiting times [19].

Finally, reducing complexity in access, triage and booking processes can free up resources for frontline care, thereby increasing the efficiency of patient flow. For example, where triage processes are used, there is evidence that simpler systems (using only two categories for “urgent” and “routine” cases, for example) may be equally as effective and more reliable than more complex multi-category systems [13].

### STAT: an alternative approach to access and triage

**Fig. 1:**
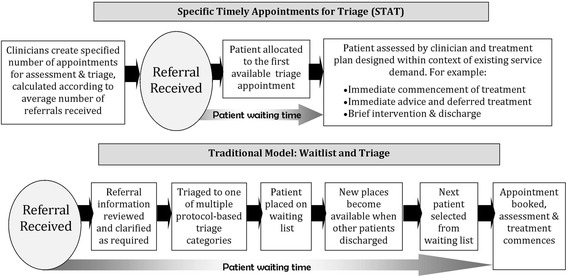
Comparison of traditional STAT and ‘Waitlist and triage’ models (originally published in http://www.publish.csiro.au/nid/270/paper/AH13033.htm, reproduced with permission^5^)

An alternative approach to the traditional ‘waitlist and triage’ approach to managing access to outpatient and community services, known as Specific & Timely Appointments for Triage (STAT), was developed using the evidence-based principles describe above. The STAT model requires clinicians to schedule a specified number of appointments for triage and assessment of new referrals in their weekly schedule, based on systematic analysis of supply and demand [5, 19, 20]. All patients are assessed in the first available time slot, and the clinician then prioritises the patient within the context of his/her caseload. Unlike traditional triage systems, clinicians are able to consider the relative priority of both new patients and those already under their care, and the system drives clinicians to seek efficiencies in patient management and make decisions in response to demand. Triage is done by people at the point of service delivery with options for management, and is flexible and based on clinical judgement. The need for complex triage protocols with high reliability becomes irrelevant. Key features of the system and comparison to a traditional waitlist are shown in Fig. 1.

A large controlled trial with a before and after design of a community rehabilitation program, found that STAT reduced waiting time by 40 % [19]. Treatment times and patient outcomes were not adversely affected by the STAT model and the results were sustained, without additional staffing, over the 6 month post intervention period of the trial. A qualitative analysis of 32 in-depth semi-structured interviews showed that the model was well received by staff and patients [5]. A further evaluation using action research methods also showed improvements in patient flow when the STAT model was applied to an outpatient physiotherapy department [21]. The success of both trials suggests that the model is well suited to single discipline or multi-disciplinary services that treat patients over a series of appointments, allowing service providers to exploit flexibility in scheduling a mixture of new assessments and review appointments.

This project aims to find out if this evidence-based alternative to the traditional use of triaged waiting lists reduces waiting time for a variety of community health and outpatient services, with no additional ongoing costs and without adversely affecting patient care.

## Methods/research design

### Research design

The research design is a Stepped Wedge Cluster Randomised Controlled Trial (SWCRCT), to evaluate the effectiveness and economic impact of the STAT model when compared to the traditional waitlist system. The SWCRT design involves sequential introduction of the STAT intervention to eight clusters (participating sites) in random order.

**Fig. 2:**
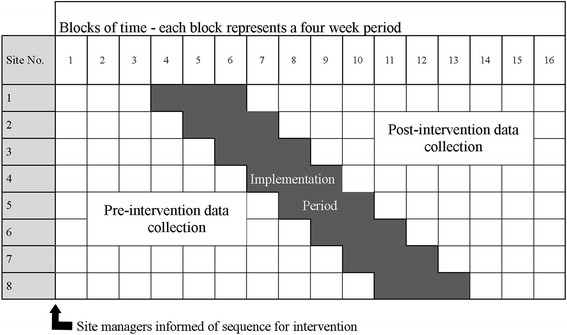
Cluster stepped wedge randomised controlled trial design

Pre-intervention data will be collected at all sites for a minimum of 12 weeks, progressing by 4 week increments in the order of intervention for the 8 sites (see Fig. 2). Each service will have a 12 week period in which to implement the STAT model, with post intervention data collection commencing at the end of this period and continuing until all services have had a minimum 12 week post intervention period.

### Inclusion criteria

The trial will include eight outpatient or community health services that:

1. Provide services to patients in the eastern suburbs of Melbourne within the structure of a single large metropolitan health care network (Eastern Health)
2. Have a waitlist for patients referred to the services
3. Typically provide their service over a series of appointments (rather than offering a single appointment for the purpose of a clinical opinion or diagnosis)
4. Do not appear to have a significant imbalance between supply and demand. That is, average waiting times may be long and demonstrate weekly, monthly or seasonal fluctuations, but the mean waiting time has not increased substantially over the previous two years.
5. Have the consent of health service management to participate in the trial.

The services that are anticipated to meet these criteria, and are therefore potential clusters for inclusion in the trial, are primarily expected to fall within one of the following groups: outpatient allied health services (such as occupational therapy, physiotherapy, speech pathology or counseling services); and specialist multidisciplinary clinics, managing conditions such as continence disorders, chronic pain, cognitive impairment or falls.

### Randomisation and masking

Following agreement from health services management to participate in the project, 8 sites will be randomised in a sequence of 1 to 8 for the purpose of order of implementation of the intervention, using a computerized random number generator (www.randomization.com). The randomization sequence will be prepared by a member of the research team not involved in the recruiting of sites to the project. This researcher will inform the project manager, who will, in turn, communicate the sequence for intervention to the managers of the participating services at the beginning of the pre intervention phase of the trial (Fig. 2).

### Intervention

**Fig. 3:**
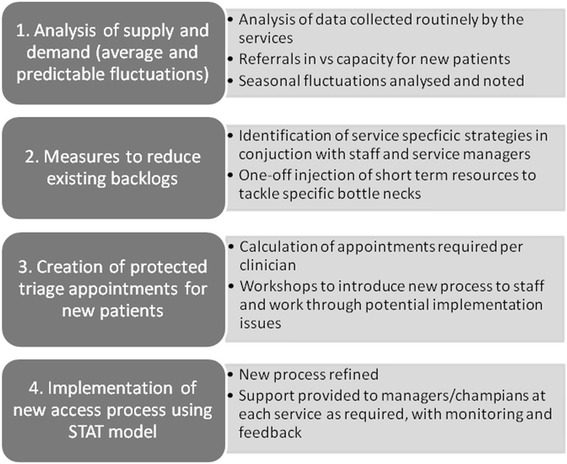
Key components of the intervention

The key components of the intervention are described in Figs. 2 and 3. Each component will be facilitated by a project officer, using workshops with staff and individual support to key clinicians as required. A small budget (capped at the equivalent of the salary budget for each service for 4 weeks) will also be made available to each service to support reduction of the existing backlog of patients on the waiting list prior to introducing the STAT model.

### Data sources

Data from all patients referred to the participating sites during the trial period will be included in service level analyses. These routinely collected data will be drawn from health service databases and supplemented with retrieval of missing data from individual medical records when required. A waiver of consent has been granted to access this routinely collected data.

A subgroup of 40 consecutive patients at each site in the pre and post intervention periods (*n* = 640) will be invited to complete health utilisation, health-related quality of life and patient satisfaction measures at admission to the service and after 12 weeks. These measures will be administered by project personnel, face to face at admission and either face to face or by telephone at 12 weeks. Individual written informed consent will be obtained for collection of these data. Participants who lack capacity to provide consent (including children and patients with moderate to severe cognitive impairment) will be included provided they have a responsible person able to provide consent on their behalf. Carers/guardians will provide health economics data on behalf of the patient, and will provide health-related quality of life data from their own perspective. Interpreters will be used as required so as not to exclude potential participants based on language.

### Outcomes measures

#### Primary outcome measure

The primary outcome measure will be waiting time, measured as the time in days from the date of referral to the date of the first face to face appointment with a clinician for the purpose of assessment/initial treatment (Table 1).

**Table 1:** Outcome measures for cluster randomised controlled trial

		Sample		Data collection points	
Outcome	Source	All patients presenting to service	Consecutive subgroup (*n* = 640)	On admission	12 weeks post admission
Primary Outcome:					
Days from referral to first appointment	Routinely collected health services data	✓		✓	
Demographic Data (age, sex, diagnosis, usual residence, referral reason/source)	Routinely collected health services data	✓		✓	
Secondary Outcomes:					
Number & type of appointments received and days from first to last appointment	Routinely collected health services data	✓			✓
Health Related Quality of Life (AQOL-8D)	Prospective survey		✓	✓	✓
Hospital admissions within 6 months of referral as a marker of adverse events	Routinely collected health services data	✓			✓
Patient/carer satisfaction with service	Prospective survey		✓		✓
Number of patients discharged without being seen and DNA rates	Routinely collected health services data	✓			✓
Individual/household health utilisation data, covering the periods (1) from referral until 1st visit and (2) 3 months following first visit.	Prospective survey		✓	✓	✓
Waiting list snapshot (number of patients on waiting list, median wait time)	Routinely collected health services data	✓		End of pre-Intervention period; beginning and end of post-intervention period	

#### Secondary outcome measures

Health service utilisation outcomes (collected for all patient commencing care with a participating service within the trial period) include: the number and nature of occasions of service (home visit versus clinic, discipline of service provider, and individual versus group); number of non-attendances; the number of patients referred to the service but discharged without receiving an appointment; the proportion discharged from the service at 12 weeks; the number of patients on the waiting list for each service (expressed also as a proportion of the average monthly capacity of the service) at the end of the pre intervention period, and the beginning and end of the post intervention period; and admissions to any of the three acute hospital sites within the health network within the first 6 months following the first face to face appointment will be audited from health service records as an indicator of health services use.

Health related quality of life (HRQoL) will be measured (on a subgroup of 40 consecutive patients at each site) using the Assessment of Quality of Life (AQOL-8D), a validated measure covering 8 domains (independent living, happiness, mental health, coping, relationships, self worth, coping, pain and senses) and a global utility score [22]. A Resource Utilisation Questionnaire designed specifically for the project will be used to measure the impact of the health condition for which the patient is receiving treatment on income, burden of formal and informal care, and health services utilisation. A patient satisfaction questionnaire adapted from the Goldstein Physical Therapy Patient Satisfaction Questionnaire [23] will be administered at the 12 week follow up to measure satisfaction with the health service comprising: 12 questions answered on a 5 point Likert scale; and two open ended questions about sources of satisfaction and dissatisfaction; and the Net Promoter Score, a rating on a 5 point scale of the likelihood of the patient recommending service to others [24].

### Analysis

#### Sample size estimation

- Total sample
  The targeted sample size for the SWCRCT is calculated by taking into account the intra-cluster correlation coefficient (ICC), the expected effect size, and the desired power (80 %) of the study as well as the number of steps, the number of baseline measurements, and the number of measurements between steps [25]. The calculation estimates that 2,496 participants (approximately 26 admissions per site per 4 week block of data collection, resulting in a minimum of 312 participants per site) are needed to detect a mean difference with small to medium effects size in waiting time at 5 % level of significance, power of 80 % and an ICC of ρ = 0.01 [26].

The sample size calculation is based on conservative estimates of the effect size detected in the pilot trial (δ = .65) which is consistent with similar effect sizes observed in studies of Advance Access in general practice settings [27].

b) Sample for individual patient data: a sub-group of the total sample
  The sub group of patients (*n* = 640) invited to complete HRQoL, resource utilisation and satisfaction questionnaires represents approximately 25 % of the total sample. Based on available data of comparable HRQoL scales evaluated across 11 studies [28] with a standardised response mean of 0.39 that was likely to be clinically significant for a comparable quality of life scale, this sample size would have greater than 95 % power to detect a difference in the utility score based on the AQOL-8D.

### Statistical analysis

The effect of STAT on the primary outcome (waiting time) will be analysed with a multiple multi-level random-effect regression model, which allows for cluster effects [26]. It will allow all identified factors (including patient characteristics such as age, sex and primary diagnosis) to be entered into the model simultaneously as covariates. The secondary outcomes will be analysed using a similar method to determine whether implementation of STAT was achieved at the expense of other aspects of service delivery, patient satisfaction or health-related quality of life. All analyses will be based on the intention to treat principle.

Cost analyses will be undertaken from two perspectives; that of the health system, and from the patient perspective. The cost of providing each service will be established over the trial period and will include the cost of practitioners, capital and overheads and administrative costs, and any additional costs of implementing the STAT model. Costing at the service level will enable cost per output unit to be measured, for example the average cost of a single service, the average cost of an outpatient episode by clinic type, and comparative efficiency measures such as the ratio of inputs to service output to be estimated. Cost analysis from the patient perspective will include the cost per patient visit, the cost per episode, the opportunity cost of time, the costs of additional health services utilised during the episode of care and the opportunity cost of any related informal care.

## Discussion

The STAT model is an example of an evidence-based approach to demand management that relies on direct allocation of services to patients rather than placement of new referrals on a waiting list. It encourages constant modification to the supply of a service to keep up with demand, is based on key principles that have been shown to improve patient flow in a range of health settings, and has been shown to be effective in community rehabilitation in a large controlled trial [19]. This is the first time that this approach will have been tested in a fully powered stepped wedge cluster randomised trial including an economic analysis, incorporating a range of differing outpatient and community health services that share key features. This paper outlines the study protocol for this trial to establish whether the STAT model of access and triage can work beyond small, well controlled pilot settings and be implemented on a large scale across multiple outpatient and community services. Secondary outcomes will determine whether the model is cost-effective, and impacts on other aspects of service delivery, patient satisfaction, or health-related quality of life.

The SWCRCT design is favoured over a traditional randomised controlled trial due to the intervention of interest being at the service level, and the high likelihood of contamination if patients were individually randomised to intervention or control groups within services [29]. The SWCRCT also has advantages over other cluster randomised controlled trial designs. It allows all participating services to receive the intervention, thereby reducing concerns associated with withholding a potentially useful intervention [29, 30]. At the same time it also provides the opportunity for discontinuation or modification of an intervention that is found to be leading to adverse outcomes at the initial sites, before replicating the situation at others. Furthermore, the sequential manner in which the intervention is introduced in a SWCRCT will increase feasibility of the project, given that the transition to the STAT model involves considerable planning and behaviour change at each site.

The intervention involves significant organisational change, and adherence to known principles for effective change management is an important element of this trial. The STAT model will be introduced in accordance with change management literature [31] and factors known to facilitate patient flow interventions [18], including those affected by the change in the planning and implementation, providing opportunities for those involved to provide feedback and contribute to problem solving, and identification of champions within each service who will assist in facilitating change [31, 32].

Addressing the existing backlog of patients waiting for services is a necessary pre-requisite in introducing the STAT model. The model is designed to maintain flow at the rate of demand, but this can only result in consistent and sustainable reductions in waiting time if there is an initial ‘one-off’ effort to address the needs of patients currently on the waiting list. Gradually working down the backlog over time has been achievable in previous trials of the STAT model [19, 21], but the specific timelines associated with the SWCRCT design require an approach that will lead to more rapid reductions in the existing waiting list at each site at the commencement of the implementation period. For this reason a small budget has been allocated to provide financial support to assist services to transition to the STAT model, including reduction of the backlog of patients currently on the waiting list. It is not anticipated that these funds will cover the cost of providing full services to all patients waiting. However, it will be available for targeted measures such as a temporary increase to an aspect of the service that is the source of a bottleneck, and is consistent with change management literature that recommends the provision of adequate resources to support change processes.

This STAT model has the potential to become a highly effective, generalisable model that will have substantial health benefits for patients and organisations delivering outpatient health services. If successful, the project will provide a significant body of evidence for a model that can significantly reduce waiting times, describe features of services that are likely to be successful in use of the model, and provide the necessary tools to support implementation in other settings. Improving access through reducing or eliminating waiting for outpatient services not only has the potential to reduce physical and psychological costs of waiting, but also ensures more resources can go directly into providing services rather than administrative processes associated with managing waiting lists. If this trial is successful, it will equip providers of outpatient and community services with an evidence-based, practical model of access that can substantially reduce waiting time with subsequent benefits for both efficiency of health systems and patient care.

## Abbreviations

AQOL-8D, Assessment of Quality of Life-8D; HRQoL, health related quality of life; ICC, Intra-cluster correlation coefficient; STAT, Specific Timely Appointments for Triage; SWCRCT, stepped wedge cluster randomised controlled trial
